# Mr. Bush, on Hernia Umbilicalis

**Published:** 1808-10

**Authors:** Francis Bush

**Affiliations:** Member of the Royal College of Surgeons, London. Frome


### To the Editors of the Medical and Physical Journal.

GENTLEMEN,

THE disease recognized by the terms Exomphalos, and Hernia Umbilicalis, is one of frequent occurrence, and sometimes is productive of considerable trouble to the practitioner.

In my remarks I shall confine myself chiefly to the cure of this disease in the infant subject, as surgeons are, I believe, pretty well agreed that umbilical hernia is a disease scarcely admitting of radical cure in the adult. However, if it be possible to effect it, the method now submitted to the public appears most likely to forward that event. For the cure of exomphalos in the young subject, two methods have been recommended. The ligature applied round the teguments, so as to produce sloughing; the protruded visceral contents being previously returned into the abdomen. And the application of a compress to the navel, supported by a bandage applied round the body. It may not be incorrect, just to notice the merits and defects of the two plans of cure.

The former has had its advocates amongst the ancients, and some of the moderns appear to favour it; some French surgeons have used it with considerable success, and we are taught to believe, there is no danger in this method of treatment; but whoever considers the operation of this remedy, must be well aware that it produces considerable inflammation, and we know that to excite inflammation in membranes lining the cavities of the abdomen, the thorax, &c., is not free from danger; it likewise often happens that inflammation once produced on such parts, runs to an extent at once dangerous to the patient, and harrassing to the practitioner. But we will suppose the ligature is productive of no inflammation beyond what we are taught to expect, it *must* be attended with severe *pain* for ten days or longer, from which irritation it would not be at all unlikely that convulsions, and sometimes even death itself, might ensue.

To the latter method, the only objection appears to be the difficuly of fixing a bandage in such amanner, as to allow freedom of motion to the patient, and at the same time to keep the parts constantly reduced. Various ingenious contrivances have been proposed for this purpose, but these have all been liable to considerable deviations, from the exact exact position they should retain to effect the intention of their application: consequently, the cure has been tedious, and has required considerable attention to accomplish it. However, incomplete as it is, this method is in general use, being wholly devoid of both danger and pain.

The plan now submitted to the profession, is one that has uniformly succeeded in a great number of cases, in accomplishing a perfect cure after a few weeks application, is unattended with danger, easy in its application, requires but little attention, and is not expensive.

I first take a piece of adhesive plaster, about 2 ½ inches square; then, the bottom of a pill box, a half crown, or any substance of the same form, in size proportioned to the protrusion, over which I wrap several slips of lint, as thin and round at the circumference, and as convex at the centre of the substance used, as possible, to compress the parts more certainly, and to render its application the easier to the patient; then, the protruded parts are carefully returned into the abdomen, the plaster applied, its centre directly over the navel, and the compress folded as above, exactly on the centre of the plaster. I then take a circular adhesive plaster, large enough to cover the compress, with which I fix it, and over it another and another, each something larger than the preceding, so as to form a conical pad, the point of which presses on the rupture; the whole is then secured by a belt of adhesive plaster, bound with moderate tightness round the body. This belt will require to be renewed once every week, or ten days, but not oftener if the plaster be properly adhesive. For this purpose I have generally used a strip of calico three or four inches wide, and long enough to bind twice round the body, covered with emplastrum thuris, with the addition of a small quantity of resina flava; this I have found to stick sufficiently well, nor have I ever known it to produce the slightest irritation of the skin, or the least inconvenience to the patient. This bandage possesses one advantage over every other before used, in not being liable to the smallest variation of position.

I am, &c.

FRANCIS BUSH,

Member of the Royal College of Surgeons, London.

*Frome, August* 16, 1803.

